# Impact of Dose-Escalated Chemoradiation on Pathological Complete Response in Patients with Locally Advanced Rectal Cancer

**DOI:** 10.3390/cancers16183170

**Published:** 2024-09-16

**Authors:** Carolina Domingo-Boluda, Diego Dualde, Teresa Taberner-Bonastre, Miguel Soler, Fernando López-Campos

**Affiliations:** 1 Hospital Universitario La Ribera (HULR), 46600 Alzira, Spain; taberner_mte@gva.es (T.T.-B.); soler_mig@gva.es (M.S.); 2 Hospital Clínico Universitario de Valencia, 46010 Valencia, Spain; di.dualdeb@comv.es; 3 Hospital Universitario Ramón y Cajal, Genesis Care Hospital Vithas La Milagrosa, 28034 Madrid, Spain

**Keywords:** locally advanced rectal cancer, neoadjuvant, chemoradiotherapy, radiotherapy dose escalation, pathological complete response

## Abstract

**Simple Summary:**

Approximately 40% of all diagnoses of rectal cancer in Spain are locally advanced. A multimodal treatment is needed, and one of its pillars is radiotherapy, with a classic dose scheme of up to 50–50.4 Gy concomitantly with oral capecitabine or 5-fluorouracil. Dose escalation is being explored to achieve higher rates of a pathological complete response, but the optimal dose and its impact on the outcomes are unclear. Our study aimed to evaluate the percentage of complete pathological responses obtained with an intensified neoadjuvant radiotherapy treatment scheme. We confirmed in a homogeneous population (49 patients treated with standard radiotherapy vs. 50 patients treated with dose-escalated radiotherapy) higher rates of a pathological complete response with a dose of up to 54 Gy concomitantly with oral capecitabine. Neoadjuvant radiotherapy intensification is useful for specimen sterilization and should be offered to selected patients.

**Abstract:**

Locally advanced rectal cancer requires a multimodal treatment. Radiotherapy is being explored for intensification to improve the rates of pathological complete responses (ypCR rates) which are correlated with better outcomes. This study reports a comparison between standard versus escalated doses in a preoperative scenario. The ypCR rates, toxicity, postoperative complications, and disease-free and overall survival at 5 years are described. From 2012 to 2019, 99 patients were analyzed retrospectively: standard arm (mean of 47.5 Gy) vs. dose-escalated arm (mean of 54.3 Gy). All patients were treated with 3DRT in 25 fractions, with concomitant capecitabine and surgery performed according to the total mesorectal excision principles in both arms. The ypCR was reported using the “College of American Pathologist grades”; the gastrointestinal (GI) and genitourinary (GU) toxicity was reported using the “Common Terminology Criteria for Adverse Events” (CTCAE 4.0). The ypCR rates were higher in the dose-escalated group (25% vs. 10.64%; *p* = 0.07), with a lower rate of non-treatment response (61.36% vs. 38.64%; *p* = 0.11). No statistical differences between the arms were found in terms of the oncological outcomes, postoperative complications (*p* = 0.15), second surgeries (*p* = 0.62), or deaths (*p* = 0.62). The CTCAE acute GI and GU toxicity were grade I or II in both arms. Our study presents a long-term follow-up in comparative cohorts.

## 1. Introduction

Colorectal cancer (CRC) is the most common neoplasm in both sexes in Spain and is the second leading cause of specific death, only behind lung cancer. It is estimated that, by 2024, 44,294 new cases will be diagnosed, of which 29,648 will be colon and 14,646 will be rectal, with the average age for a CRC diagnosis being around 70 years [1]. Hereditary factors have little impact on the population’s incidence (less than 5% of CRC cases), and most are sporadic tumors with a multifactorial etiology. Throughout the disease, 20–25% of patients will develop metastasis, and the overall mortality is around 40–45% [2].

The TNM classification at diagnosis is the most important prognostic factor [3]. Other factors related to survival include the number of lymph nodes resected after surgery (a minimum of 12 [4]), the response to neoadjuvant treatment, the involvement of the radial resection margin, venous and perineural invasion [5,6], obstructive and/or perforated tumors, a grade of GIII [7,8], the age, carcinoembryonic antigen (CEA) values above 20 ng/mL [9], and the patient’s status performance [10,11].

The classic treatment scheme includes preoperative chemoradiotherapy, as it reduces pelvic recurrences, controls micrometastases, increases the complete response rates to treatment, and improves the overall survival by around 10% [12,13,14,15,16,17]. The standard scheme of RT is conventional fractionation (1.8–2 Gy/fraction/day) of up to 50–50.4 Gy combined with capecitabine (CPC) or 5-fluorouracil (5-FU). Complete pathological responses with this treatment scheme are around 20%, and tumor regression is achieved in 40–80% of cases [18,19,20].

Surgery is the main curative treatment, and the technique of choice is a low or ultralow anterior resection with total mesorectal excision (TME), as it preserves the function of the sphincter complex. It is usually performed between 6 and 8 weeks after neoadjuvant treatment [21].

Adjuvant chemotherapy is administered in high-risk stage II or stage III patients, despite having achieved a complete clinical response, and it has shown benefits for the DFS. The standard scheme is based on fluoropyrimidines (with or without oxaliplatin) by extrapolation from the data of the ADORE study [22,23]; however, assessing the risk/benefit should be individualized.

Since 2020, following the publication of the RAPIDO study results [24], there are new strategies for neoadjuvant treatments in rectal cancer.

In the RAPIDO trial, patients received nine cycles of FOLFOX (5-fluorouracil + oxaliplatin) or six of CAPOX (capecitabine + oxaliplatin) in a neoadjuvant setting and a short cycle of RT (5 × 5 Gy) before surgery. A ypCR rate was observed in favor of the experimental arm compared to the arm with 50.4 Gy conventional fractionation + CPC (28% vs. 14%; *p* < 0.0001). The improvement in the local recurrence (LR) and disease-free-survival (DFS) was not statistically significant: the LR was 7.2% vs. 4.3% (*p* = 0.20) and the DFS was 28.5% vs. 24.4% (*p* = 0.34).

The total neoadjuvant treatment strategy was explored in another four prospective trials: Polish-II [25], TIMING (the only non-randomized one) [26], FOWARC [27], GCR-3 [28], and in a meta-analysis [29]. This strategy improves the rates of complete pathological responses and has proven to be a surrogate factor for the DFS [30].

“Watch and wait” or observation after a complete clinical response is not a standard strategy. The decision should arise within a clinical trial or be agreed upon by the multidisciplinary committee in the selected cases. The recommended follow-up in these cases, according to the Habr-Gama et al. protocol [31], should be carried out with anamnesis every 6–10 weeks during the first two years, every 3 months during the third year, and every 6 months from the fourth year, including the following in all of them: a rectal examination, a rigid colonoscopy, and the determination of CEA levels. In the OPRA study, it was established that patients should undergo a rectal examination and a flexible proctosigmoidoscopy every 4 months during the first 2 years from the time of the response evaluation with magnetic resonance imaging (MRI) every 6 months in those first 2 years. During the following 3 years, a rectal examination and a flexible sigmoidoscopy should be repeated every 6 months [32].

Our objective was to retrospectively assess the impact of clinical, histological, and outcome parameters from dose intensification radiotherapy concomitantly with oral capecitabine, and compare the rates of pathological complete responses between the two different arms of the treatment dose.

## 2. Materials and Methods

Based on a prospective database, a retrospective study was conducted on all patients treated consecutively between June 2012 and December 2019 with neoadjuvant radiotherapy for locally advanced rectal cancer.

Since our study was single-institutional, our series were treated homogeneously with a consistent and uniform pathological specimen analysis. Clinicians made the decision on dosimetry jointly with physicians in a daily meeting session based on organ-at-risk limitations.

### 2.1. Sample Population

All patients were evaluated by the multidisciplinary committee for digestive tumors, which included a pre-treatment study consisting of a physical examination, a rectal examination, a colonoscopy, a biopsy, a thoracoabdominal–pelvic computerized tomography (CT) scan, abdominopelvic MRI, a chest X-ray, a complete blood count, and blood tests with tumor markers.

The inclusion criteria were as follows: patients over 18 years old diagnosed with American Joint Committee on Cancer stage II–III rectal adenocarcinoma (classified according to the 6th edition) [33] who had received at least one dose greater than 50.4 Gy (considered standard in classic neoadjuvant chemoradiation schemes).

The exclusion criteria were as follows: patients who ultimately did not undergo surgery (due to refusal or a high surgical risk), synchronous tumors at diagnosis, and those with a history of other neoplasms.

**Figure 1:**
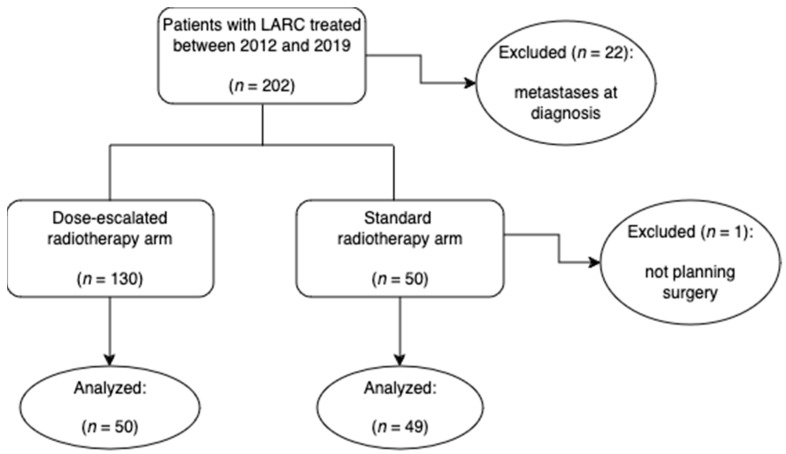
CONSORT diagram of the study: the implementation of 3DRT fields by the Radiophysics and Radiological Protection Department made it possible to treat the majority of the patients with a dose-escalated protocol from 2012 onwards (see Section 2.2.2 below).

Patients with LARC were evaluated 1:1 to either a standard treatment arm consisting of preoperative chemoradiation (mean of 47.5 Gy) or to a dose-escalated radiotherapy arm (mean of 54.3 Gy), as a CONSORT diagram shows in Figure 1.

### 2.2. Treatment Protocol

#### 2.2.1. Neoadjuvant Chemotherapy

Only 4 patients, those with stage cT4 and/or cN2 at diagnosis and a positive extramural invasion, received neoadjuvant chemotherapy. The most commonly used regimens were 1 to 4 cycles of CAPOX. In all cases, the chemotherapy concomitant with radiotherapy was oral CPC at 825 mg/m^2^ every 12 h on all days that radiotherapy was administered.

#### 2.2.2. Radiotherapy

The patients were immobilized in the supine position with an indexed vacuum mattress, knee support, and arms on the chest [34].

The treatment volumes were defined according to the consensus guidelines for the contouring of gross, clinical, and planning tumor volume structures in anorectal cancer [35].

The upper limit for tumors in the middle–upper third of the rectum was L5-S1, depending on the location of the peritoneal reflection. For tumors in the lower third, the upper limit of the irradiation volume was S1–S2.

The dose limits applied were based on the specific organ at risk as follows: bladder (V40 < 50%, Dmax < 65 Gy), femoral heads (V50 < 5–10%, Dmax < 42 Gy), and small intestine (defined in our center as the abdominal cavity 1 cm above the PTV; V40 < 150cc or as close as possible).

The technique used was the “box” type with 3D conformal radiotherapy (0°, 90°, 180°, and 270° fields), with a multileaf collimator when the dose went up to 50.4 Gy (standard arm). In addition to the “box” technique, lateral fields were modified and oblique fields were added in the boost phase to spare the small bowel when the dose went up to 54 Gy (dose-escalated arm). The planning system used for the dose calculation was Pinnacle^®^ from Oracle^®^. The treatment was performed on a linear accelerator (2100 CD MLC from Varian^®^) using 6 and 18 MV photons as required by the patient’s anatomy. Daily positioning verifications were carried out for all the patients using MV radiographic imaging.

All patients diagnosed with CRLA in the Radiotherapy Oncology Department of HULR were planned to receive the accelerated boost with the escalated dose protocol, but the dosimetry was approved at the discretion of the clinician in charge based on compliance with the restrictive doses for the organs at risk. Currently, there is a majority consensus on contouring the small intestine by outlining the abdominal cavity. This approach is more homogeneous, reproducible, and safer when it comes to correctly predicting the side effects on this organ at risk. By consensus, in our center, the abdominal cavity was contoured and used as the dose limit to the small intestine, with V40 < 150cc or as close to this value as possible. If the objective was not reached, the patient was immediately planned for treatment with standard doses.

We differentiated two treatment groups: the dose-escalation group, with a mean of 54.3 Gy (IQR 43.4–60.76 Gy) in 25 fractions over the tumor, and the group that received standard radiotherapy doses, with a mean of 47.5 Gy (IQR 43.2–50.4 Gy) and the same number of total fractions.

#### 2.2.3. Planned Surgery

The response was evaluated using CT or MRI after a minimum interval of about 6 weeks. Only in 8 patients could neither of these tests be performed. In all the patients, a TME was performed. For lower rectal tumors close to the sphincter complex, an abdominoperineal resection (APR) was performed; however, for those with a sufficient margin, a low anterior resection was carried out. Hartmann’s procedure was performed in the patients with a high surgical risk or as a temporary treatment to later perform a reconstruction in patients with a prolonged surgical time.

#### 2.2.4. Adjuvant Chemotherapy

Postoperative chemotherapy was optional based on the postoperative pathological features and a distant risk factor dissemination. The most commonly used regimens were XELOX and CPC in monotherapy, or both, when oxaliplatin was withdrawn from XELOX due to poor tolerance/toxicity. Seventy percent (*n* = 35) of the patients in the dose-escalation arm received adjuvant chemotherapy, with a mean of 4.6 cycles (IQR: 2–8); 87.23% (*n* = 41) of the patients in the standard arm received a mean of 4.8 cycles (IQR: 3–6).

#### 2.2.5. Response

During chemoradiotherapy, the patients were evaluated weekly; a complete blood count and basic biochemistry were requested biweekly.

As for acute radiation-induced toxicity, data on diarrhea, cystitis, radiodermatitis, abdominal pain, and proctitis were collected and classified according to the CTCAE, version 4.0. Likewise, chronic GI and GU toxicity were recorded starting from the first year after completing treatment, with serial records at the third and fifth years.

The usual follow-up for patients was every 3–4 months during the first two years, every 6 months until the fifth year, and then annually until discharge, which could be up to 10 years. For complementary tests, the recommendations of the ESMO guidelines [11] were followed.

To evaluate the tumor response to chemoradiotherapy, the four-category tumor regression grading (TRG) system developed by the American Joint Committee on Cancer and the College of American Pathologists (AJCC/CAP) was used [36]: TRG 0 (complete response; no tumor cells), TRG 1 (near-complete response; isolated cells or small groups of isolated cells), TRG 2 (partial response; residual tumor with evident regression, but more groups of cells), and TRG 3 (poor or no response; extensive residual tumor with no evidence of regression). The following variables were also collected: lymphovascular, perineural, and extramural vascular invasion and the status of the resection margins (proximal, distal, and circumferential) according to AJCC/TNM R0, R1, and R2, based on whether the resection was clear, microscopically involved, or macroscopically involved, respectively.

Postoperative complications were defined by surgical, infectious, cardiovascular, or other causes, and the need for surgical re-intervention in these patients.

Surgical mortality was defined as that occurring within the first 30 days after the intervention. For the survival analysis, the following variables were defined:

Overall survival (OS): calculated as the interval of time from the date of the surgery, considered the definitive curative intent treatment, to the patient’s death from any cause. Patients who were alive at the end of the study were censored at the time of the last follow-up visit.

Disease-free survival (DFS): calculated as the time from the date of the surgery to the date of the detection of the first disease relapse and/or death, whichever event occurred first. Patients who were alive and disease-free were censored at the time of the last follow-up visit.

### 2.3. Statistical Analysis

As measures of central tendency and dispersion for quantitative variables, the median and interquartile range were described, respectively. For categorical variables, frequency statistics such as the absolute and relative percentages were used.

We evaluated the association between two categorical variables using the chi-square method, and if an expected frequency was less than 5, Fisher’s exact test was used. In contrast, the association between quantitative variables was evaluated using Student’s *t*-tests or an analysis of variance (ANOVA), and continuous variables were analyzed with the Mann–Whitney U test. The precision and statistical significance in all cases were at a 95% confidence interval (CI) and a significance level of 5% (*p*-value < 0.05), respectively.

For the analysis of the overall survival and disease-free survival, estimations were made for the entire series and comparisons were made between patients with different responses to neoadjuvant treatment using the Kaplan–Meier method. The precision and statistical significance in all cases were at a 95% confidence interval (CI) and a significance level of 5% (*p*-value < 0.05), respectively.

To build the models, a univariable analysis was conducted, where variables with a *p*-value > 0.10 were selected for inclusion in the multivariate model. In the multivariate analysis, only variables with a *p*-value < 0.05 were considered significant. The data were analyzed using the Stata^®^ statistical software, version 14.2 (StataCorp LP, College Station, TX, USA).

## 3. Results

### 3.1. Characteristics of the Patients

From 2012 to 2019, 99 patients were retrospectively analyzed (standard group: 49 and dose-escalation group: 50), with a median follow-up of 5.93 years (IQR: 0.03–11.33). The characteristics of the patients by treatment group are shown in Table 1. The proportions between the treatment arms were similar, indicating that both arms were well balanced. The variables that influenced the achievement of pathological complete responses were analyzed in the present study.

### 3.2. Surgery

The surgical procedure par excellence for preserving the sphincter complex was the low anterior resection, which was performed on the majority of patients. The second most performed intervention was the abdominoperineal resection. In only one patient from the dose-escalation group, a curative surgery was not performed; instead, due to a ypCR and the advanced age of the patient, a local excision was decided upon. This patient was not included in the disease control and survival analyses, as they did not meet the criteria for definitive treatment.

There were no differences between the groups in terms of all the postoperative complications (*p* = 0.15), second surgeries (*p* = 0.62), or deaths during postoperative recovery (*p* = 0.62). The most common complication was anastomotic dehiscence or leakage in the dose-escalation group, which involved two more re-interventions in this group than in the standard-dose group (*p* = 0.09). This was followed by paralytic ileus and intra-abdominal collection as the second most common complication in the dose-escalation group and the first in the standard group. Two patients in the standard group and one in the dose-escalation group died within 30 days following the intervention. The causes of death in the patients in treatment arms were as follows: two cases in the standard arm—one due to fecal peritonitis with no possibility of repairing the anastomotic dehiscence and another due to a cerebrovascular accident. In the dose-escalation arm, there was one case of death due to persistent paralytic ileus. The surgical complications experienced by the patients can be found in Table 2 (Clavien–Dindo classification).

### 3.3. Pathological Response and Downstaging

The dose-escalated group obtained more than double the number of pathological complete responses vs. the standard group (25% vs. 10.64%; *p* = 0.07), showing a trend towards significance, and a lower rate of no response to the treatment (38.64% vs. 61.36%; *p* = 0.11). The rate of responders and non-responders in the standard group was similar. Downstaging was similar in both groups. The variables related to our treatment protocol and the response by group are shown in Table 3.

### 3.4. Acute and Chronic Toxicity

The completion of neoadjuvant treatment in both groups was satisfactory and a similar number of patients had interruptions in both groups. The most common cause of interruption was grade III diarrhea, followed by proctitis. There were twice as many interruptions associated with chemotherapy, since these patients continued radiotherapy without CPC until resolution. The cases of radiotherapy interruption (two patients in each arm) corresponded to the non-administration of the last sessions due to grade III diarrhea.

Grade II acute GI toxicity (according to the CTCAE, version 4.0) was lower in the standard group (16.33% vs. 24%; *p* = 0.06). Only four patients (8%) in the dose-escalation group experienced grade III acute gastrointestinal toxicity, and none experienced this in the standard group. The grade II acute GU toxicity was lower in the standard group than in the dose-escalation group (6.12% vs. 8%; *p* = 0.39). No patient experienced a higher grade of toxicity. The grade I GI toxicity at 3 years was lower in the standard group (12% vs. 18%; *p* = 0.25). Only two patients (4.55%) had grade II and one patient (2.27%) had grade III in the dose-escalation group. Most patients in both groups were asymptomatic at 3 years in terms of GU toxicity (97.62% vs. 95.45%; *p* = 1.00).

### 3.5. Univariable and Multivariate Analyses

The variables analyzed were as follows: age, sex, tumor distance from the anal margin, tumor size (cT) and number of positive lymph nodes (cN), histological grade and CEA at diagnosis, tumor distance to the mesorectal fascia (measured by MRI at diagnosis), and the interval of time from neoadjuvant therapy to surgery.

In the univariable analysis, we found that, for achieving a complete pathological response, a smaller tumor size (cT2) was the most favorable factor *(p* = 0.01). Although the RT dose did not reach statistical significance, its OR (Table 4) showed that, with dose escalation, more than double the complete pathological responses can be obtained compared to the standard dose (25% vs. 10.64%), with the results not being statistically significant due to the sample size limitation (*p* = 0.07). Since only one variable reached statistical significance, no further multivariate analyses were performed.

### 3.6. Control and Survival

**Figure 2:**
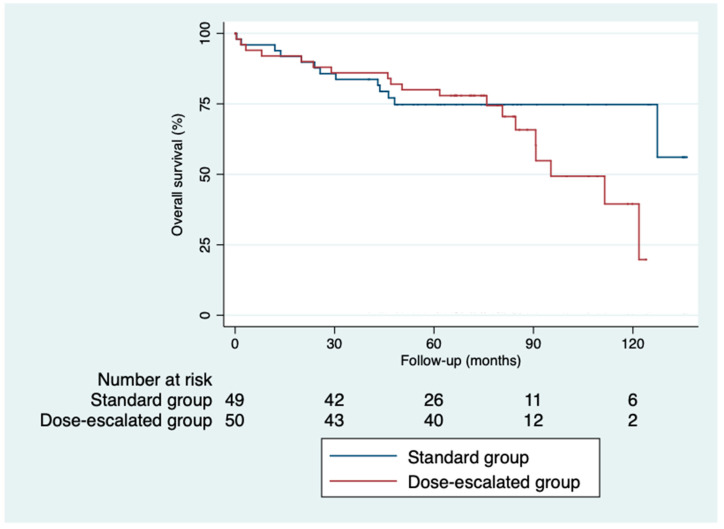
Kaplan–Meier curves for overall survival.

**Figure 3:**
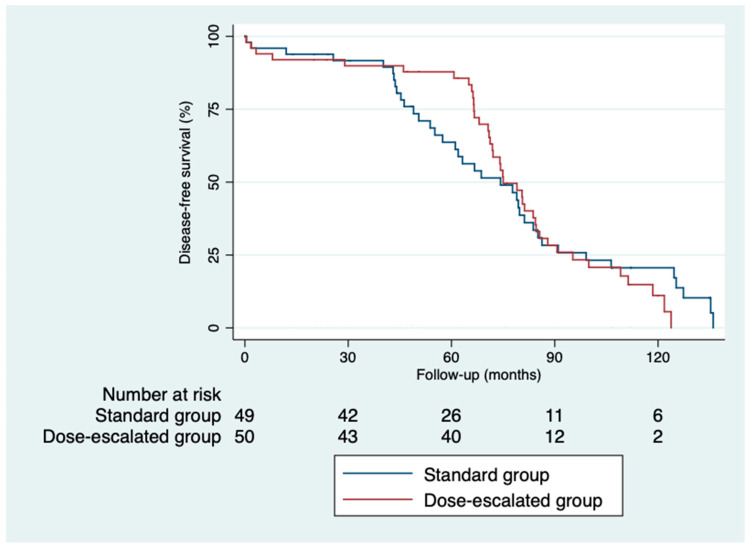
Kaplan–Meier curves for disease-free survival.

**Figure 4:**
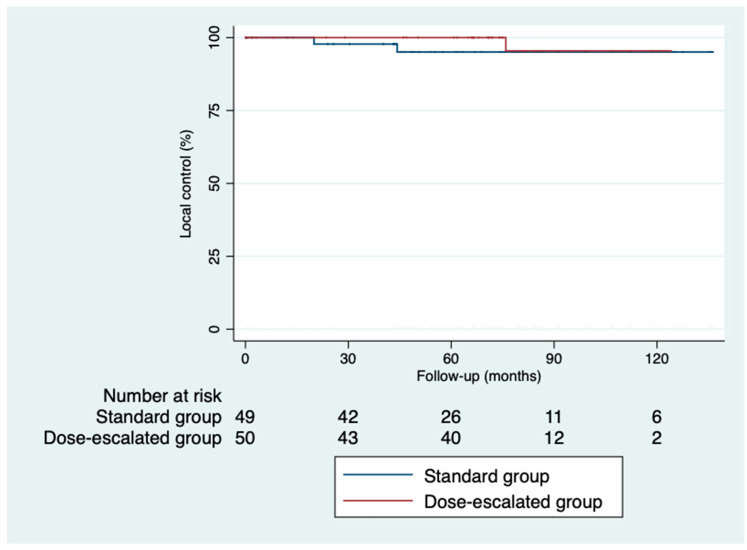
Kaplan–Meier curves for local control.

The 5-year disease-free survival was higher in the dose-escalation group than in the standard group (87.6% vs. 64%; *p* = 0.82), as was the 5-year overall survival (81.6% vs. 74.6%; *p* = 0.32) (Figure 2 and Figure 3). However, we did not find a benefit in terms of the outcomes, despite achieving higher rates of pathological complete responses with a median follow-up of 5.93 years (IQR: 0.03–11.33). The local control was similar in both groups (Figure 4). Only three local recurrences were documented in the entire series: one in the dose-escalation group, found outside the irradiation field, and two recurrences in the standard group, located within the irradiation field. Nine distant recurrences were reported in each group. Regarding the patients who achieved a ypCR, only two of them died from rectal cancer. Concerning recurrences in the patients with a ypCR, only two patients had a distant recurrence, and none had a locoregional recurrence.

## 4. Discussion

Intensifying the neoadjuvant treatment by increasing the radiotherapy dose in patients diagnosed with locally advanced rectal cancer (LARC) achieved ypCR rates of 25% in our series, which were similar to those reported in the literature in dose-escalation studies [37,38] and higher than those reported by the standard radiotherapy regimen [18,19,20], without a relevant increase in the overall postoperative complications or toxicity.

The preoperative treatment of locally advanced rectal cancer with chemoradiotherapy was established as the standard to reduce tumor volume and achieve R0 surgeries, as well as lower the rates of complications and toxicity compared to administering radiotherapy postoperatively [21]. Since then, new neoadjuvant treatment regimens have been sought to increase the disease-free survival and overall survival. The ypCR is considered an independent factor for increasing the local control (LC) and disease-free survival (DFS) [39,40,41,42]. Our treatment regimen was based on the Xelac arm (Xeloda and Conocomitant boost RT) in the Interact Trial [43] as a reference regimen compared to Xelox (Xeloda–Oxaliplatin and standard RT). Our primary objective was to compare the ypCR in a group of patients treated with integrated dose escalation of the tumor versus standard doses of radiotherapy. Unlike in the Interact Trial, where the boost was administered twice a week for 5 weeks, in our case, it was administered from the beginning at a dose of 2.16 Gy/day, reaching 54 Gy instead of the 55 Gy of the Interact Trial, although this represents an equivalent biological dose (BED). The ypCR rate achieved was 25% versus 10.64% in favor of dose escalation, with no significant difference, but a trend toward significance (*p* = 0.07). Both the short- and long-term toxicity was acceptable, indicating that our regimen is safe.

To our knowledge, the first study to evaluate the pathological response rate by increasing the RT dose in American patients to 52.5 Gy in conjunction with intravenous 5 FU was published in 2000 [44]. Subsequently, in 2006, the same group published the long-term results for 54 patients, reporting the same RT dose, but concurrent with oral capecitabine, demonstrating a safety profile. They achieved an 18% ypCR rate and a 59% downstaging rate. The first meta-analysis published in 2014 gathered studies conducted to date with 3D irradiation techniques using dose escalation via a linear accelerator or, in some cases, high-dose-rate brachytherapy; none used the IMRT technique. The meta-analysis concluded that increasing the dose to ≥60 Gy in LARC increases the ypCR rate with an acceptable acute toxicity, although randomized controlled trials with larger patient samples are needed [37]. A second, more recent meta-analysis from 2021 included studies using intensity-modulated radiation therapy (IMRT) with an accelerated boost (between 54 and 60 Gy) that were not included in previous studies. It concluded that a dose of ≥54 Gy is associated with high ypCR rates close to 25%, achieved with a dose boost of between 54 and 60 Gy with IMRT, all without increasing the risk of grade III acute toxicity. However, a clear dose–response relationship was not identified in the regression analysis. The optimal boost dose has not been determined, and data on late toxicity and long-term oncological outcomes are lacking in the literature [38]. Other studies using an integrated boost with IMRT are those by Hernando-Requejo [45] and Jin-luan Li [46]—reporting ypCR rates close to 31%—and Lupatelli [47]. Except for these studies, we do not know any others that have reported higher ypCR rates.

From the univariable analyses, we found that the variable influencing the achievement of a ypCR was the clinical tumor size (cT). Patients with a favorable profile for better tumor regression are those with tumors not presenting deep invasion. A recently published retrospective study similar to ours by Qi Zhang [48] found that the CEA value and a surgical interval >8 weeks are variables influencing higher pathological response rates.

Our study had some limitations. The retrospective nature introduced a potential bias in the selection and data analysis. The decision to administer the dose-escalated protocol was made by the clinician in charge, and we could not find information about the response to the treatment in eight patients. We reviewed 3DRT planning because we had more follow-up information for these cohorts. Since the introduction of advanced techniques (IMRT), no more patients have been treated with 3DRT planning in our institution, and that explains the sample size of this study. The *p*-value of 0.09 for suture failures in anastomoses is not negligible. Confounding factors such as the surgical team with practicing surgeons, the type of suture material used, and intraoperative complications that might explain this higher rate were not considered. A review is planned in the near future with a IMRT dose-escalated boost with the same scheme as that reported in the present study. Since our study was single-institutional, our series were treated homogeneously with consistent and uniform pathological specimen analysis, and it offered data with a long follow-up, which are some of the major strengths of the study.

## 5. Conclusions

In our study, we found a trend towards higher ypCR rates with neoadjuvant 3DRT intensification compared to the standard scheme. Although no statistical differences were shown, intensification is useful for specimen sterilization and should be offered to selected patients (tumors without deep invasion in our cohorts). Well-designed randomized and controlled trials are needed to obtain conclusive data.

## Figures and Tables

**Table 1:** Characteristics of the patients.

	Standard Arm N = 49	Dose-Escalated Arm N = 50	*p*-Value
Age (years) ≥65 y <65 y	Median = 66.7 (41.1–89.6) 29 (40.82%) 20 (59.18%)	Median = 66.2 (40.9–86.7) 26 (48%) 24 (52%)	0.36 0.47
Gender (female/male)	F = 20 (40.82%) M = 29 (59.18%)	F = 16 (32%) M = 34 (68%)	0.36
Tumor distance from internal anal margin (mm)	Mean = 53.88 0–50 = 13 (26.53%) 51–100 = 24 (48.98%) ≥ 101 = 12 (24.49%)	Mean = 58.6 0–50 = 12 (24%) 51–100 = 25 (50%) ≥ 101 = 13 (26%)	0.53 0.96
cTNM stage	IIA = 6 (12.77%) IIB = 2 (4.26%) IIIA = 4 (8.51%) IIIB = 14 (29.79%) IIIC = 21 (44.68%)	IIA = 5 (10%) IIB = 3 (6%) IIIA = 10 (20%) IIIB = 7 (14%) IIIC = 25 (50%)	0.24
Histological grade Grade 1 Grade 2 Grade 3	39 (81.25%) 7 (14.58%) 2 (4.17%)	37 (75.51%) 10 (20.41%) 2 (4.08%)	0.84
Pretreatment CEA ^1^ ≥ 5 ng/mL (yes/no)	Yes = 23 (52.27%) No = 21 (47.73%)	Yes = 21 (42%) No = 29 (58%)	0.32
Pretreatment mesorectal fascia invasion (yes/no)	Yes = 34 (69.39%) No = 15 (30.61%)	Yes = 30 (61.22%) No = 19 (38.78%)	0.40

^1^ CEA: carcinoembryonic antigen.

**Table 2:** Type of complications.

Complications	Standard Arm N = 49	Dose-Escalated Arm N = 50	*p*-Value
None	26 (53.06%)	23 (46%)	0.48
Wound	1 (2.04%)	3 (6%)	0.62
Abdominal	7 (14.29%)	9 (18%)	0.62
Urinary tract	3 (6.12%)	1 (2%)	0.36
Anastomosis: Leak Fistula Ischemia	4 (8.16%) 1 (2.04%) -	10 (20%) - 1 (2%)	0.09
Stoma	1 (2.04%)	2 (2%)	1.00
Cardiovascular	4 (8.16%)	1 (2%)	0.20
Others	3 (6.12%)	-	-
Clavien–Dindo classification Grade I Grade II Grade IIIa Grade IIIb Grade IVa Grade IVb Grade V	7 (30.43%) 4 (17.39%) 4 (17.39%) 5 (21.74%) 1 (4.35%) - 2 (8.70%)	7 (25.93%) 1 (3.70%) 9 (33.33%) 8 (29.63%) - 1 (3.70%) 1 (3.70%)	0.39

**Table 3:** Variables related to treatment and response.

	Standard Arm N = 49	Dose-Escalated Arm N = 50	*p*-Value
Completion of treatment Radiotherapy Chemotherapy	45 Gy: 24 (48.98%) 50 Gy: 10 (20.41%) 50.4 Gy: 13 (26.53%) Interruptions: (43.2 Gy): 2 (4.08%) Interruptions: 4 (8.16%)	53.5 Gy: 6 (12%) 54.25 Gy: 41 (82%) 60.76 Gy: 1 (2%) Interruptions: (43.4 Gy): 2 (4%) Interruptions: 5 (10%)	0.51
Surgery Time interval (weeks) from chemoradiation to surgery	Saving-sphincter: 33 (67.35%) Definitive stoma: 15 (30.61%) Hartmann: 1 (2.04%) <8 weeks: 11 (22.45%) ≥8 to <9 weeks: 7 (14.29%) ≥9 to <10 weeks: 14 (28.57%) ≥10 weeks: 17 (34.69%)	Saving-sphincter: 33 (67.35%) Definitive stoma: 15 (30.61%) Hartmann: 1 (2.04%) Hemicolectomy: 1 (2%) <8 weeks: 8 (16%) ≥8 to <9 weeks: 9 (18%) ≥9 to <10 weeks: 15 (30%) ≥10 weeks: 18 (36%)	1.00 0.85
CAP ^1^	ypCR = CAP 0: 5 (10.64%) CAP 1: 16 (34.04%) CAP 2: 13 (27.66%) CAP 3: 13 (27.66%) Unknown: 2	ypCR = CAP 0: 11 (25%) CAP 1: 16 (36.36%) CAP 2: 13 (29.55%) CAP 3: 4 (9.09%) Unknown: 6	0.07
CAP ^1^-grouped (responders vs. non-responders)	CAP 0 + 1: 21 (44.68%) CAP 2 + 3: 26 (55.32%)	CAP 0 + 1: 27 (61.36%) CAP 2 + 3: 17 (38.64%)	0.11
Downstaging	Yes: 40 (81.63%) No: 9 (18.37%)	Yes: 44 (88%) No: 6 (12%)	0.38

^1^ CAP: College of American Pathologists.

**Table 4:** Univariable analysis.

	*p*-Value	Odds Ratio	IC 95% Inferior	IC 95% Superior
Age (years)	0.79	1.00	0.96	1.06
Gender (male vs. female)	0.64	1.27	0.40	4.00
Tumor location:	0.82			
Middle vs. inferior	0.87	0.90	0.24	3.41
Superior vs. inferior	0.71	1.31	0.31	5.60
cT3 vs. cT2	0.01			
cT3 vs. cT2	0.01	0.16	0.04	0.66
cT4 vs. cT2	0.78	0.83	0.23	3.07
cN stage:	0.46			
cN1 vs. cN0	0.39	2.07	0.39	11.11
cN2 vs. cN0	0.96	1.05	0.19	5.82
cTNM (reference: stage 0)	0.67			
IIB	0.55	2.5	0.12	50.44
IIIA	0.25	4	0.38	42.37
IIIB	0.47	2.35	0.23	24.09
IIIC	0.72	1.5	0.16	13.92
Histologic grade:				
Grade 2 vs. grade 1	0.96	1.04	0.26	4.14
Pretreatment CEA ^1^ ≥ 5 ng/mL (yes vs. no)				
	0.25	0.51	0.16	1.64
Pretreatment mesorectal fascia invasion (yes vs. no)				
	0.36	1.73	0.51	5.85
Weeks from chemoradiation to surgery (reference: <8)	0.78			
≥8 to <9	0.86	1.21	0.15	9.76
≥9 to <10	0.34	2.32	0.41	12.96
≥10	0.52	1.76	0.32	9.71
Dose of RT:				
>50.4 Gy	0.11	2.48	0.79	7.77

^1^ CEA: carcinoembryonic antigen.

## Data Availability

The data presented in this study are available in an anonymized form from the corresponding author upon request.
